# The interaction of Kinesin-1 with its adaptor protein JIP1 can be regulated via proteins binding to the JIP1-PTB domain

**DOI:** 10.1186/1471-2121-14-12

**Published:** 2013-03-04

**Authors:** Tomoko Satake, Karin Otsuki, Yumi Banba, Jun Suenaga, Hisashi Hirano, Yuko Yamanaka, Shigeo Ohno, Syu-ichi Hirai

**Affiliations:** 1 Department of Molecular Biology, Yokohama City University Graduate School of Medicine, Yokohama 236-0004, Japan; 2 Molecular Medicine and Informatics Doctoral Program, Yokohama City University Graduate School of Medicine,Yokohama 236-0004, Japan; 3 Department of Supramolecular Biology, International Graduate School of Arts and Sciences, Yokohama City University, Yokohama 230-0045, Japan; 4 Department of Biology, Wakayama Medical University School of Medicine, Wakayama 641-0011, Japan

**Keywords:** Kinesin-1, JIP, Cargo adaptor, PTB domain, Transport

## Abstract

**Background:**

The regulatory mechanisms of motor protein-dependent intracellular transport are still not fully understood. The kinesin-1-binding protein, JIP1, can function as an adaptor protein that links kinesin-1 and other JIP1-binding “cargo” proteins. However, it is unknown whether these “cargo” proteins influence the JIP1–kinesin-1 binding.

**Results:**

We show here that JIP1–kinesin-1 binding in Neuro2a cells was dependent on conserved amino acid residues in the JIP1-phosphotyrosine binding (PTB) domain, including F687. In addition, mutation of F687 severely affected the neurite tip localization of JIP1. Proteomic analysis revealed another kinesin-1 binding protein, JIP3, as a major JIP1 binding protein. The association between JIP1 and JIP3 was dependent on the F687 residue in JIP1, and this association induced the formation of a stable ternary complex with kinesin-1. On the other hand, the binding of JIP1 and JIP3 was independent of kinesin-1 binding. We also show that other PTB binding proteins can interrupt the formation of the ternary complex.

**Conclusions:**

The formation of the JIP1–kinesin-1 complex depends on the protein binding-status of the JIP1 PTB domain. This may imply a regulatory mechanism of kinesin-1-dependent intracellular transport.

## Background

Kinesin-1, also known as conventional kinesin, is a member of the Kinesin super family proteins (KIFs). KIFs move along microtubules (MTs) by ATP hydrolysis and are essential for the transport of a variety of cargos such as protein complexes and membranous organelles [1,2]. In neurons, kinesin-1 plays a role in axonal transport of synaptic vesicles and mitochondria [1,2]. Kinesin-1 is a heterotetramer composed of two kinesin heavy chains (KHC or KIF5) and two kinesin light chains (KLC). The KHCs contain an N-terminal motor domain and a C-terminal tail region, which is required for the interaction with KLCs. The KLCs are required for the interaction with cargo proteins. Efficient intracellular transport depends on a dynamic association-dissociation cycle of motor and cargos that may involve certain regulatory molecules [1]. Kinesin-1 often employs specific adaptor proteins to carry cargos [2]. As such, the interaction between kinesin-1 and adaptor proteins can potentially serve as a regulatory point for motor-cargo assembly. However, this potential mechanism has not been directly investigated.

JNK interacting protein (JIP) 1 binds to the KLC through its C-terminal kinesin-binding site [3]. JIP1 also binds to a variety of other proteins through its JNK binding domain (JBD) and phosphotyrosine binding (PTB) domain [4-9]. Therefore, JIP1 may function as an adaptor that physically connects JIP1-binding cargo proteins and kinesin-1 to enable the transport of specific proteins. Genetic analyses of *Drosophila* axonal transport have revealed the physiological significance of JIP1 in supporting kinesin-1-dependent intracellular vesicle transport [4,5]. The binding mode of JIP1 to potential cargo proteins has been precisely analyzed. The JBD is required for interaction with JNK [6], while the PTB domain is required for interaction with various PTB domain binding proteins, including amyloid precursor protein (APP), apolipoprotein E receptor 2 (ApoER2), p190RhoGEF, dual leucine zipper bearing kinase (DLK), and JIP3 (JSAP1) [7-11]. The PTB domain binds to proteins containing an NPxY motif (or NxxY, NxxF) through an interaction dependent on a conserved phenylalanine residue in the PTB domain [12]. The corresponding phenylalanine residue of JIP1, F687, is required for interaction with the NPTY motif of APP and the NEAF motif of p190RhoGEF [8,13]. The PTB domain of JIP1 also binds to proteins which do not have typical NPxY motif including DLK and JIP3. These observations suggest a critical regulatory role for JIP1 in kinesin-1-dependent intracellular transport, and the importance of JIP1-binding proteins in regulating the formation of the JIP1–kinesin-1 complex. However, the effects of JIP1-binding proteins on the formation of the JIP1–kinesin-1 complex have not been fully determined.

In this study, we tested the significance of JIP1 binding proteins for the formation of the JIP1–kinesin-1 complex in mammalian cells. We demonstrated that conserved amino acid residues in the PTB domain, including F687, but not the JBD of JIP1 enhance the formation of a stable complex with kinesin-1, while the C-terminal residues show an absolute requirement for this interaction. We then identified another kinesin-1 binding protein, JIP3, responsible for the F687-dependent enhancement of the formation of the JIP1–kinesin-1 complex. We further analyzed the molecular basis of the enhancement of JIP1–kinesin-1 complex formation. The results not only suggest a regulatory role of JIP3 in the formation of the JIP1–kinesin-1 complex, but also suggest a possible regulatory mechanism mediated by JIP1-binding proteins that bind to the JIP1-PTB domain.

## Results

### Formation of the JIP1–kinesin-1 complex in Neuro2a cells is independent of the JIP1-JBD and cellular JNK activity

**Figure 1:**
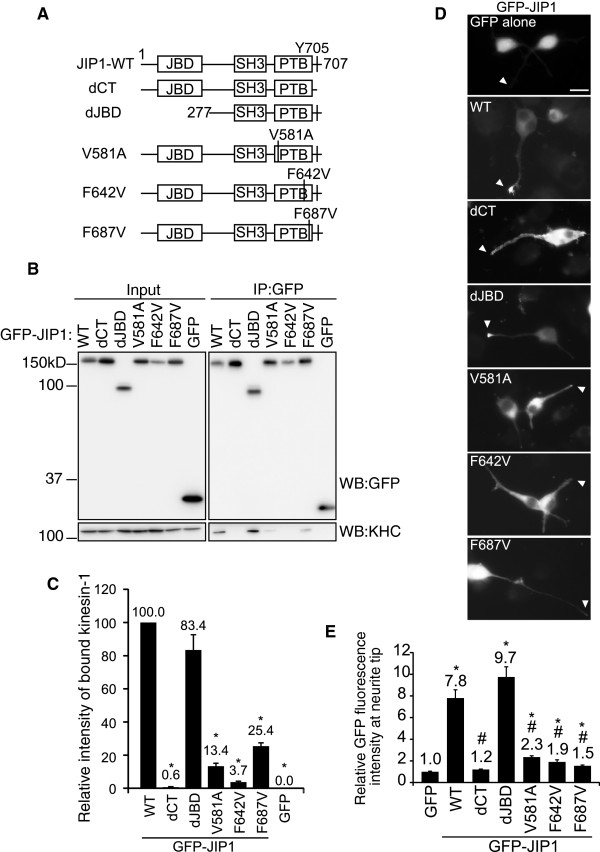
**Formation of the JIP1–kinesin-1 complex in Neuro2a cells is dependent on the JIP1-PTB domain but not JIP1-JBD. **(**A**). Schematic illustration of the JIP1 constructs used in this study. The N-terminal 276 residues were deleted in dJBD. The C-terminal 4 residues were deleted in dCT. V581, F642 and F687 were substituted by A or V in V581A, F642V and F687V. JBD: JNK binding domain; SH3: src homology 3 domain; PTB: phosphotyrosine binding domain; Y705: required tyrosine for kinesin-1 binding. These constructs are GFP- or TAP-tagged at their N-termini. (**B**). Lysates prepared from Neuro2a cells expressing GFP-JIP1 constructs or GFP alone were immunoprecipitated with anti-GFP antibody and analyzed by western blotting (WB) with the indicated antibodies. *Input*, cell lysate used for the immunoprecipitation assay. *IP:GFP*, immunoprecipitated proteins. (**C**). Quantification of kinesin-1 binding by the GFP-JIP1 constructs in (**A**). Kinesin-1 binding was normalized to the amount of precipitated GFP-JIP1. *: p < 0.001 compared with GFP-JIP1-WT. Error bars indicate ± SEM. n > 3. (**D**). Differentiated Neuro2a cells were transfected with expression vectors encoding the GFP-JIP1 constructs shown in (**A**) or GFP alone. Arrowheads indicate the neurite tips of transfected cells. Scale bar = 20 μm. (**E**). The neurite tip localization of GFP fusion proteins is shown by the relative fluorescence (tip/shaft ratio) of GFP, compensated by the value of free GFP. *: p < 0.001 compared with GFP. #: p < 0.001 compared with GFP-JIP1-WT. Error bars indicate ± SEM. n = 117 (GFP), 203 (WT), 183 (dCT), 213 (dJBD), 184 (V581A), 64 (F642V), 157 (F687V). These were derived from three independent experiments.

To examine the requirement of JIP1 binding proteins for the association between JIP1 and kinesin-1, we made a series of deletions or amino acid substitutions in the JBD and PTB domains of JIP1 (Figure 1A). The C-terminal 4 residues, which include the kinesin-1 binding site [3], were deleted in the dCT mutant, which served as a negative control. The mutated JIP1 proteins were tagged with GFP at their N termini and transiently expressed in differentiated Neuro2a cells. The association between the JIP1 mutants and kinesin-1 was estimated by an immunoprecipitation assay using anti-GFP antibody (Figure 1B and C). The results demonstrated that GFP-JIP1-WT and GFP-JIP1-dJBD showed comparable binding activity to kinesin-1, while binding activity was almost completely absent from GFP-JIP1-dCT (Figure 1B and C). Control GFP did not bind to kinesin-1. It has been reported that GFP-tagged JIP1 localizes to the neurite tips of cultured neuronal cells when the C-terminal kinesin-1 binding site is intact [3]. We confirmed the localization of GFP-JIP1 to neurite tips in a kinesin-1 binding site-dependent manner (Figure 1D, WT and dCT). This suggests that we can evaluate the association between JIP1 and kinesin-1 *in vivo* by monitoring the subcellular localization of JIP1. Thus, the localization of WT and mutant GFP-JIP1 at neurite tips was evaluated as the relative fluorescence ratio between the tips and shafts of neurites, using free GFP as a control (Figure 1E). Deletion of the N-terminal region of JIP1 that includes the JBD did not affect the localization of JIP1 to neurite tips, as expected from the binding data described above (Figure 1D, dJBD). Rather, the neurite tip localization of GFP-JIP1-dJBD was somewhat greater than GFP-JIP1-WT, although the difference was not statistically significant (Figure 1E). Because the JIP1-JBD can bind to JNK, we next tested the association between JIP1 and JNK by evaluating the co-precipitation of endogenous JNK with mutant or WT GFP-JIP1 (Additional file: 1 Figure S1A). JNK was co-precipitated with JIP1-WT, the dCT mutant and the PTB domain mutants, but not with the dJBD mutant, confirming that the dJBD mutant had lost the ability to bind JNK. Taken together, these results indicate that the JBD of JIP1 is not essential for the association between JIP1 and kinesin-1.

A possible role for JNK in the interaction between JIP1 and kinesin-1 has been proposed. In *Drosophila*, over-expression of hemipterous, an upstream kinase of the JNK ortholog, Basket, disrupts the binding of the JIP1 ortholog, APLIP1, to kinesin-1 [5]. This suggests that the binding of JIP1 to kinesin-1 depends on the activation state of JNK. However, the dJBD mutant of JIP1 that lost the ability to bind JNK apparently retained full binding activity to kinesin-1, as described above. Further, neither inhibition nor activation of JNK affected the co-precipitation of kinesin-1 with JIP1 in Neuro2a cells (Additional file: 1 Figure S1B and C). These results are consistent with the notion that JNK activity does not affect the binding of JIP1 and kinesin-1 in mammalian systems.

### The PTB domain of JIP1 is required for complex formation with kinesin-1

We next examined the requirement of the PTB domain for the association between JIP1 and kinesin-1. Because deletion of the JIP1 PTB domain resulted in insoluble aggregate formation in Neuro2a cells, we used amino acid substitution to generate PTB domain mutants that lacked protein-binding activity (Figure 1A). By aligning the sequence of the JIP1 PTB domain with those of other proteins such as Dab1 and Shc, divergence between the N-terminal regions of the PTB domains in these proteins became apparent (Additional file: 2 Figure S2A). This suggests that the N-terminal region of the PTB domain is responsible for JIP1 specific functions. Alignment of JIP1 PTB domain sequences from various species revealed that V581 in this region is highly conserved (Additional file: 2 Figure S2B). Therefore, we substituted this amino acid with A to make the JIP1-V581A mutant, which was predicted to lose the ability to bind to some JIP1-specific binding proteins. Two conserved phenylalanine residues, F642 and F687, located in the C-terminal region of the JIP1 PTB domain are required for its binding to the NPTY motif of APP [13]. We replaced these F residues with V to make the JIP1-F642V and JIP1-F687V mutants, respectively (Additional file: 2 Figure S2B). We used these JIP1 mutants to examine the requirement of the PTB domain for the association between JIP1 and kinesin-1 using two independent methods described previously. As shown in Figure 1B and C, kinesin-1 binding to the GFP-JIP1-PTB domain mutants (V581A, F642V and F687V) was somewhat greater than to the dCT mutant or GFP, but far less than to GFP-JIP1-WT. The localization of GFP-JIP1 at neurite tips was also dependent on the PTB domain: none of JIP1-PTB domain mutants (V581A, F642V and F687V) showed obvious localization to the tips of neurites (Figure 1D). The neurite tip localizations of these mutants were greater than the dCT mutant or GFP, but far less than GFP-JIP1-WT (Figure 1E). Taken together, these results indicate that the PTB domain, in addition to the C-terminal region, contributes to the association between JIP1 and kinesin-1 in differentiated Neuro2a cells.

### Identification of JIP3 as a principal binding protein of JIP1

**Figure 2:**
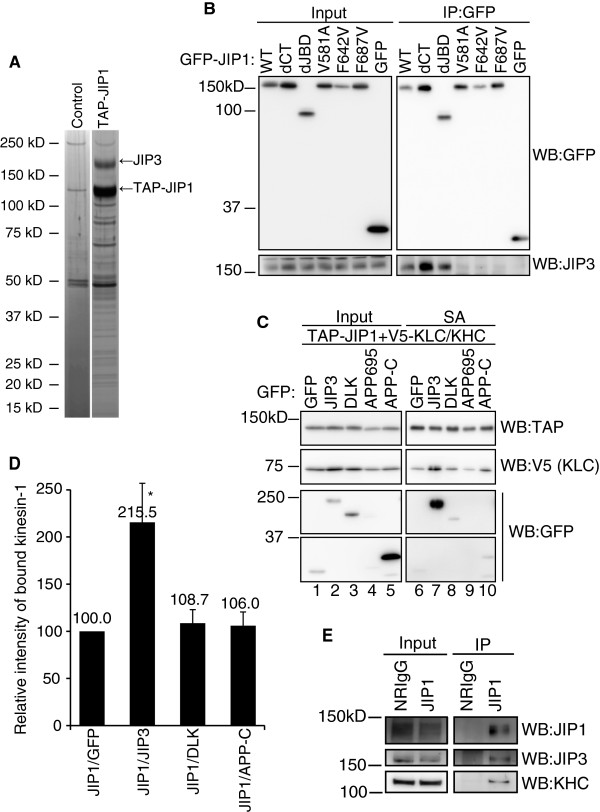
**Identification of JIP3 as a major PTB domain-dependent binding protein of JIP1. **(**A**). Neuro2a cells transiently transfected with either TAP-JIP1 or control vector were used for pull-down experiments with streptavidin sepharose (SA). The JIP1 associated polypeptides were visualized by silver staining. (**B**). Co-precipitation of GFP-JIP1 constructs and endogenous JIP3 in Neuro2a cells. The same samples as shown in Fig. 1B were analyzed by WB with the indicated antibodies. (**C**). Lysates of HEK293T cells expressing TAP-JIP1, V5-tagged kinesin light chain (V5-KLC), V5-KHC, GFP-JIP3, DLK, APP, or GFP alone were precipitated with SA and analyzed by WB with the indicated antibodies. *Input*, cell lysate used for the immunoprecipitation assay. *SA*, proteins bound to streptavidin sepharose. (**D**). Quantification of kinesin-1 (V5-KLC) binding to TAP-JIP1 in (**C**). Kinesin-1 (V5-KLC) binding was normalized to the amount of precipitated TAP-JIP1. *: p < 0.003 compared with GFP. Error bars indicate ± SEM. n > 3. (**E**). An E17 mouse brain extract was immunoprecipitated with anti-JIP1 antibody. Precipitates were analyzed by WB with the indicated antibodies.

The V581, F642 and F687 mutations in the PTB domain drastically reduced the binding of JIP1 to kinesin-1. Because the JIP1-PTB domain cannot directly interact with kinesin-1 [14], we hypothesized that a protein binds to the PTB domain of JIP1 and enhances the association between JIP1 and kinesin-1. We therefore searched for JIP1 binding proteins in Neuro2a cells. SDS-PAGE analysis of proteins co-precipitated with TAP-tagged JIP1 revealed a protein with an apparent molecular weight of ~180 kDa as a major binding partner of JIP1 (Figure 2A). This protein was identified as JIP3 by mass spectrometry (Additional file: 3 Figure S3). JIP3 has been reported to bind to the JIP1 PTB domain in vitro [11], but whether V581, F642 or F687 are required for this binding is unknown. Thus, we examined the binding between GFP-JIP1 mutants and endogenous JIP3 in Neuro2a cells by co-precipitation assays. Co-precipitation of JIP3 was observed with GFP-JIP1-WT, GFP-JIP1-dJBD and GFP-JIP1-dCT, but not with the V581A, F642V and F687V mutants or GFP (Figure 2B). These results indicate that the JIP1–JIP3 interaction in Neuro2a cells depends on V581, F642 and F687 in the JIP1 PTB domain. Accordingly, JIP3, a major JIP1-binding protein in differentiated Neuro2a cells, is a candidate protein that could enhance the association between JIP1 and kinesin-1.

To test this possibility, we performed co-precipitation assays using lysates from HEK293T cells, which did not contain detectable levels of JIP3, JIP1, and kinesin-1 proteins. Cells were transfected with expression vectors for TAP-tagged JIP1, V5-tagged KLC, and V5-tagged KHC, together with an expression vector for GFP-JIP3 or GFP. As shown in Figure 2C, the amount of kinesin-1 (V5-KLC) co-precipitated with JIP1 was significantly increased by co-expression of JIP3 (lanes 6 and 7). Because the molecular weight of V5-KHC was very close to that of TAP-tagged JIP1, we routinely detected V5-KLC when detecting TAP-JIP1 in the same gel. Notably, GFP-JIP3 was also found in the JIP1 binding fraction (Figure 2C, lane 7). These results clearly demonstrate that JIP3 can enhance the association between JIP1 and kinesin-1.

Although we were able to identify JIP3 as a major JIP1 PTB domain binding protein in differentiated Neuro2a cells, several proteins other than JIP3 have been reported as JIP1 PTB domain binding proteins in other cell systems [7-10]. These proteins include DLK and APP. DLK is a protein kinase that activates the JNK pathway [15]. DLK binding to JIP1 is mostly lost by deletion of the C-terminal part of JIP1 that includes the PTB domain [10]. APP is a vesicle-associated protein that binds to JIP1 in a manner dependent on the F642 and F687 residues of JIP1 [13]. To compare the JIP1 binding activity of these proteins, we performed co-precipitation assays using cell lysates prepared from HEK293T cells expressing GFP-fusion constructs of these JIP1 binding proteins, together with TAP-JIP1, V5-KLC and V5-KHC. Because of the low expression of full-length human APP (APP695), we used the cytoplasmic region of APP (APP-C), which contains the JIP1 binding region [7]. As shown in Figure 2C (lanes 7, 8, and 10), the amounts of GFP-DLK and GFP-APP-C in the JIP1-bound fraction were far less than GFP-JIP3, although their expression levels were rather higher (lanes 2, 3, and 5). Moreover, the expression of DLK or APP-C showed little effect on the amount of kinesin-1 co-precipitated with JIP1 (Figure 2D). These results suggest a unique function of JIP3 compared to other JIP1-binding proteins.

The association of JIP1 with JIP3 and kinesin-1 was evaluated *in vivo* by immunoprecipitation of an E17 mouse brain extract with anti-JIP1 antibody. The results clearly demonstrated that the immunoprecipitated complex containing JIP1 also contained JIP3 and kinesin-1 (Figure 2E). This result may imply the physiological significance of the association between JIP3 and JIP1.

### JIP3 enhances the association between JIP1 and kinesin-1 in Neuro2a cells

**Figure 3:**
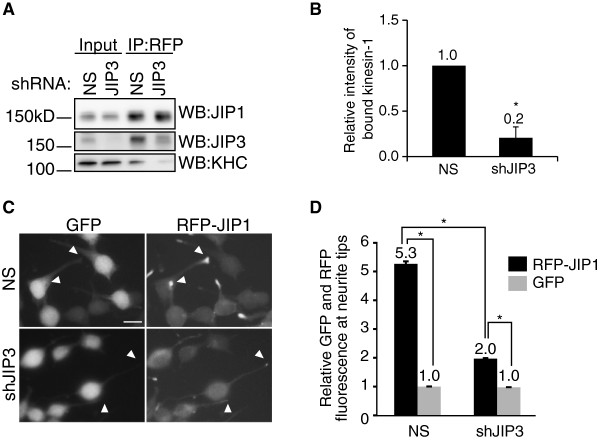
**JIP1 requires JIP3 for binding to kinesin-1 in Neuro2a cells. **(**A**). Lysates prepared from RFP-JIP1-Neuro2a cells expressing shRNA targeting JIP3 (shJIP3) or non-silencing control (NS) shRNA were immunoprecipitated with anti-RFP antibody and analyzed by WB with the indicated antibodies. *Input*, cell lysate used for the immunoprecipitation assay. IP:RFP, immunoprecipitated proteins. (**B**). Quantification of kinesin-1 binding by RFP-JIP1 in (**A**). Kinesin-1 binding was normalized to the amount of precipitated RFP-JIP1. *: p = 0.004 compared with NS. Error bars indicate ± SEM. n = 3. (**C**). Differentiated RFP-JIP1-Neuro2a cells were transfected with shRNA vectors (NS or shJIP3) containing a GFP expression cassette. Arrowheads indicate the neurite tips of transfected cells. Scale bar = 20 μm. (**D**). Quantification of the relative fluorescence of RFP-JIP1 and GFP in the same neurite tip. *: p < 0.001. Error bars indicate ± SEM. n = 200 neurites for each construct.

To test whether JIP3–JIP1 binding is essential for the association between JIP1 and kinesin-1, we investigated the effect of JIP3 knockdown. Neuro2a cells stably expressing RFP-tagged JIP1 (RFP-JIP1-Neuro2a cells) were transiently transfected with expression vectors for JIP3 shRNA (shJIP3) or non-silencing shRNA (NS). Immunoprecipitation of RFP-JIP1 followed by western blotting using anti-KHC antibody revealed that the binding of RFP-JIP1 and kinesin-1 was severely reduced by JIP3 knockdown (Figure 3A and B). We also tested whether the JIP3 knockdown altered the neurite tip localization of RFP-JIP1. Differentiated RFP-JIP1-Neuro2a cells were transiently transfected with shRNA vectors (NS or shJIP3) containing a GFP expression cassette to help identify the transfected cells. The localization of RFP-JIP1 to the neurite tip of GFP-labeled cells was significantly reduced in shJIP3-transfected cells compared with NS transfected cells (Figure 3C and D). The neurite tip localization of GFP was not observed in either the control cells or the JIP3-knockdown cells. The kinesin-1 binding and neurite tip localization of RFP-JIP1 reduced by JIP3 knockdown were almost recovered by the TAP-JIP3-WT expression without recovering the endogenous JIP3 level (Additional file: 4 Figure S4). These results indicate that JIP3 is crucial for the association between JIP1 and kinesin-1 in Neuro2a cells.

### Molecular basis for JIP1–JIP3–kinesin-1 ternary complex formation

**Figure 4:**
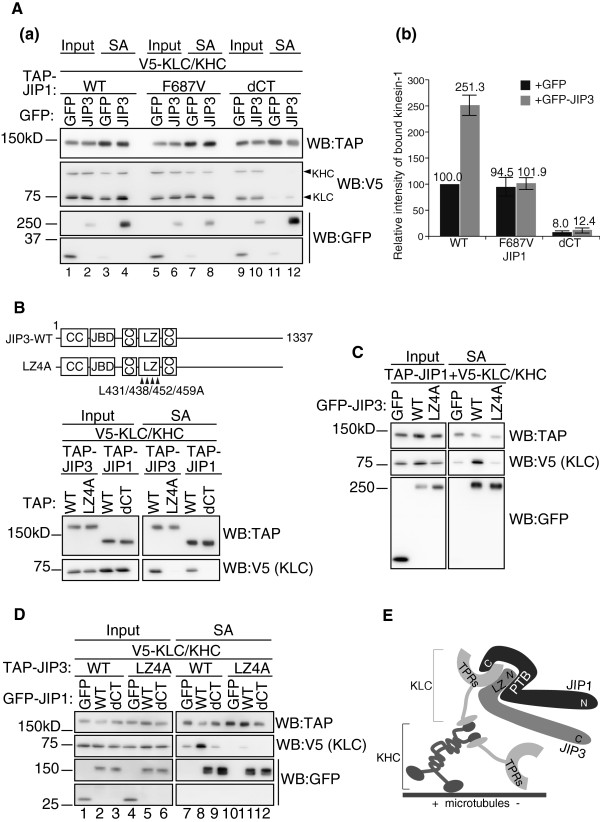
**Molecular basis for JIP1–JIP3–kinesin-1 ternary complex formation. **(**A**). Lysates prepared from HEK293T cells expressing TAP-JIP1, V5-KLC, V5-KHC and GFP-JIP3, or GFP alone, were precipitated with SA and analyzed by WB with the indicated antibodies. *Input*, cell lysate used for the immunoprecipitation assay. *SA*, proteins bound to streptavidin sepharose. Graph shows quantification of kinesin-1 (KLC) in the TAP-JIP1-bound fraction “SA”. Each value was normalized to the amount of TAP-JIP1 in the corresponding SA fraction. *: p <0.001 compared with TAP-JIP1-WT + GFP. Error bars indicate ± SEM. n = 3. (**B**). Upper: schematic illustration of the JIP3 constructs. The L residues (431, 438, 452 and 459) were substituted for A in the JIP3-LZ4A mutants. These constructs are GFP- or TAP-tagged at their N-termini. JBD: JNK binding domain; LZ: leucine zipper; CC: coiled-coil. Lower: lysates prepared from HEK293T cells expressing TAP-JIP3, V5-KLC and V5-KHC were precipitated with SA. TAP-JIP1 constructs were used as controls. (**C**). Lysates of HEK293T cells expressing TAP-JIP1-WT, V5-KLC, V5-KHC, GFP-JIP3 or GFP alone were precipitated with SA and analyzed by WB with the indicated antibodies. (**D**). Lysates of HEK293T cells expressing TAP-JIP3, V5-KLC, V5-KHC, GFP-JIP1 or GFP alone were precipitated with SA and analyzed by WB with the indicated antibodies. (**E**). A model of the JIP1–JIP3–kinesin-1 complex. JIP1 binds to the TPR domain of KLC via the C-terminal region. JIP3 also binds to the TPR domain of KLC via the LZ domain. Because JIP1 and JIP3 bind to distinct surfaces within the TPR domain of KLC [11], JIP3 binding to the JIP1-PTB domain can form a molecular complex that grasps the TPR domain and binds to the TPR domain more stably than JIP1 or JIP3 alone.

As shown in Figure 2C, the ectopic expression of JIP3 in HEK293T cells enhanced the association between JIP1 and kinesin-1. To confirm that the effect of JIP3 depends on the binding of JIP3 to the JIP1 PTB domain, we performed co-precipitation assays using the JIP1-F687V mutant, which showed negligible binding to JIP3 (Figure 2B). Although the expression of GFP-JIP3 enhanced the binding of TAP-tagged JIP1-WT to kinesin-1 (V5-KHC/KLC) (Figure 4A, lanes 3 and 4), the effect of JIP3 expression was negated by the JIP1 F687V mutation (Figure 4A, lanes 7 and 8). Concomitant binding of GFP-JIP3 was significantly reduced by the F687V mutation (Figure 4A, lanes 4 and 8). Nonetheless, in the absence of JIP3, the weak kinesin-1-binding activity of the JIP1-F687V mutant was comparable with that of JIP1-WT, indicating that the F687V mutation does not affect the basal binding capacity of JIP1 to kinesin-1 (Figure 4A, lanes 3 and 7). This basal kinesin-1 binding capacity was dependent on the C-terminal sequence of JIP1 (Figure 4A, lanes 3 and 11). These results indicate that JIP1–JIP3 complex formation via F687 significantly increases the kinesin-1-binding capacity of JIP1, compared with JIP1 alone.

We confirmed that roughly equal amounts of V5-KHC and V5-KLC were present in the JIP1 pull-down fraction (see Figure 4A). This suggests that V5-KHC and V5-KLC form the kinesin-1 holoenzyme in HEK293T cells and bind to JIP1.

We then sought to clarify why the JIP1–JIP3 complex showed such higher kinesin-1-binding activity. Because both JIP1 and JIP3 can bind to kinesin-1 [1], we tested whether the binding of JIP3 to kinesin-1 is essential for the stable binding of the JIP1–JIP3 complex to kinesin-1. To disrupt the binding of JIP3 to kinesin-1, we constructed a JIP3-LZ4A mutant by substituting four L residues in the JIP3 kinesin-1 binding site, L431, L438, L452 and L459, with A (Figure 4B upper column). These L residues are conserved between JIP3 and a highly related protein, JIP4, and are directly involved in JIP4–kinesin-1 binding [16]. As shown in Figure 4B (lower column), the JIP3-LZ4A mutant failed to bind to kinesin-1. Although the JIP1-binding capacity of the JIP3-LZ4A mutant was comparable with wild-type JIP3, the ability to enhance the binding of JIP1 and kinesin-1 was completely lost in this mutant (Figure 4B, right middle panel). This result indicates that JIP3–kinesin-1 binding, as well as JIP1–kinesin-1 binding, is essential for the stable association of the JIP1–JIP3 complex with kinesin-1 (Figure 4B). This notion is confirmed by the observation that TAP-JIP3-LZ4A fails to offset the effect of JIP3 knockdown on the JIP1–kinesin-1 binding and the neurite tip localization of RFP-JIP1 (Additional file: 4 Figure S4).

Finally, we examined whether the JIP1–JIP3 complex is stabilized by kinesin-1. As shown in Figure 4A (lanes 4 and 12) and Figure 4C, disruption of the kinesin-1 binding site in both JIP1 and JIP3 did not affect the binding of JIP1 to JIP3. Moreover, concomitant disruption of JIP1 and JIP3 binding to kinesin-1 also had no effect on the binding of JIP1 to JIP3, while the binding of kinesin-1 was completely abolished (Figure 4D, lanes 8, 9, 11, and 12). These results indicate that JIP1 and JIP3 can form a stable protein complex, even in the absence of kinesin-1.

### DLK affects the formation of the JIP1–JIP3–kinesin-1 ternary complex

**Figure 5:**
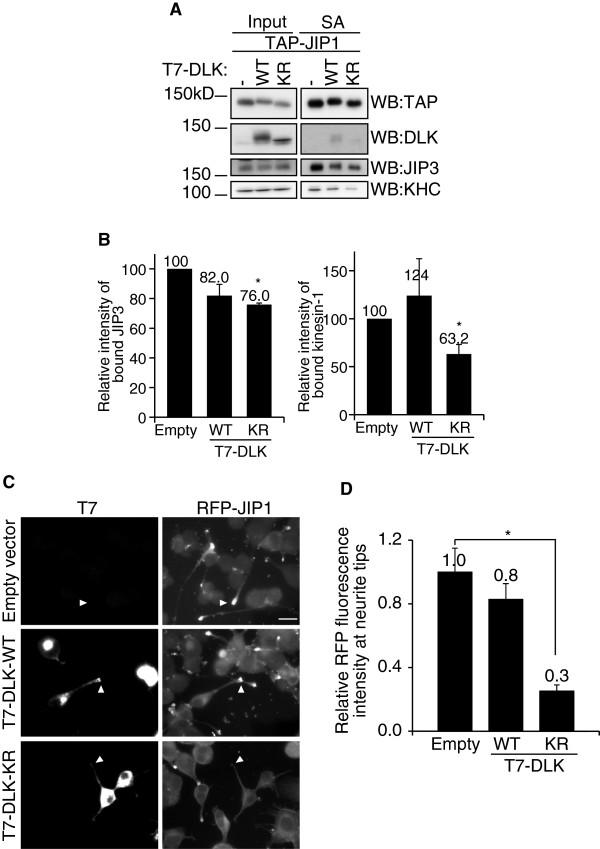
**DLK affects the formation of the JIP1–JIP3–kinesin-1 ternary complex. **(**A**). Lysates of Neuro2a cells expressing TAP-JIP1 and T7-DLK-WT, T7-DLK-KR or pUC8 were precipitated with SA and analyzed by WB with the indicated antibodies. *SA*, proteins bound to streptavidin sepharose. (**B**). Quantification of the binding of JIP3 and kinesin-1 to TAP-JIP1 in (**A**). The extent of binding was normalized to the amount of precipitated TAP-JIP1. *: p < 0.036 compared with Empty. Error bars indicate ± SEM. n = 4. (**C**). Differentiated RFP-JIP1-Neuro2a cells were transfected with expression vectors for T7-tagged DLK-WT, T7-DLK-KR, or pUC8 (empty vector). T7-DLK was detected by indirect immunofluorescence microscopy using anti-T7 antibody. Arrowheads indicate the neurite tips of transfected cells identified by phase contrast microscopy. Scale bar = 20 μm. (**D**). Quantification of the relative fluorescence of RFP-JIP1 at the neurite tip. *: p < 0.001. Error bars indicate ± SEM. n = 100 neurites for each construct.

The results presented above suggest the presence of a JIP1–JIP3–kinesin-1 ternary complex. We then tested whether the formation of the ternary complex was regulated by an additional factor, which may serve as a regulator of the JIP1–JIP3–kinesin-1 complex formation. Because the formation of the JIP1–JIP3–kinesin-1 ternary complex was highly dependent on PTB domain-dependent binding of JIP1 and JIP3, we hypothesized that other PTB domain-dependent JIP1 binding proteins would interrupt the formation of the ternary complex. If the PTB binding protein has no kinesin-1 binding activity, the binding of kinesin-1 to this protein complex will be restricted. One of the candidates for such a protein is DLK, which is reported to stably interact with JIP1 via the PTB domain, particularly when its kinase activity is suppressed [10,17]. As shown in Figure 5A and B, over-expression of a kinase-inactive mutant of DLK (DLK-KR) in Neuro2a cells did partially suppress JIP1–JIP3 binding and JIP1–kinesin-1 binding. It also suppressed the neurite tip localization of JIP1 (Figure 5C and D). A significant suppressive effect was observed only with the kinase-inactive mutant of DLK, while the effect of wild-type DLK over-expression on these binding events was not significant. This may reflect the complex regulation of the protein-binding activity of JIP1 and DLK by their phosphorylation status [17]. In any case, these results suggest a potential regulatory role for DLK in the formation of the JIP1–JIP3–kinesin-1 ternary complex.

## Discussion

In this study, we investigated the contribution of JIP1 binding proteins to the interaction between JIP1 and kinesin-1. Although the binding of JNK to the JBD of JIP1 showed no significant effect on the kinesin-1 binding, the binding of JIP3 to the PTB domain of JIP1 enhanced it significantly. DLK, another JIP1-PTB domain binding protein, did not show such an effect. However, the over-expression of kinase-inactive DLK perturbed the formation of the JIP1–JIP3–kinesin-1 ternary complex, suggesting that DLK might compete with JIP3 for the JIP1-PTB domain. Taken together with the reported function of JIP1 as a cargo adaptor [3], these results suggest that the potency of the interaction between JIP1 and kinesin-1 depends on which protein binds to the JIP1-PTB domain.

JIP1 and JIP3 were originally identified as JNK binding proteins that function as a scaffold for the JNK activating protein kinase cascade in mammalian cells [18,19]. While JIP1 is now recognized as a cargo adaptor that connects cargo and kinesin-1 [3], the relationship between these distinct JIP1 functions remained unclear. It has been reported that the activation of JNK leads to the unloading of kinesin-1 from MTs [20]. However, the contribution of JIP1 or JIP3 to this process is ambiguous. A previous study in *Drosophila* suggested the possibility that JIP1–kinesin-1 binding is disrupted by JNK activation via an unidentified JNK target [5]. Because the *Drosophila* JIP1 ortholog, APLIP1, lacks the JBD, the effect of JNK may not be dependent on JIP1–JNK binding. We show here that the binding of JIP1 to kinesin-1 is not affected by the binding of JNK to JIP1, regardless of JNK activity, in mammalian cells. JIP1–JIP3 binding was also independent of JIP1–JNK binding (see Figure 2B and Additional file: 1 Figure S1A). We still cannot rule out the possibility that JIP3–JNK binding affects JIP1–JIP3 binding or JIP1–kinesin-1 binding. However, such scenarios may be unlikely because it has been reported that deletion of the JBD in JIP3 does not affect JIP3 transport in neural cells [21], and because the JIP1–JIP3 interaction in Neuro 2a cells was independent of JNK activity (see Additional file: 1 Figure S1B). Taken together, JNK seems to be a cargo of the JIP1–JIP3–kinesin-1 complex rather than a regulator of JIP1-JIP3-kinesin-1 complex formation.

In contrast to the dispensability of the JIP1 JBD, the PTB domain is essential for kinesin-1 binding by JIP1 in differentiated Neuro2a cells. We identified JIP3 as a major PTB-dependent JIP1 binding protein that was largely essential for the neurite tip localization and kinesin-1-binding of JIP1. JIP1 or JIP3 bound to kinesin-1 via their kinesin-1-binding motifs, without forming a JIP1–JIP3 complex. However, this “basal” kinesin-1 binding activity was significantly enhanced by JIP1–JIP3 complex formation, which was totally dependent on the JIP1-PTB domain. A previous study showed that distinct regions of the TPR domains in KLC are responsible for JIP1 and JIP3 binding [11]. Therefore, JIP1 and JIP3 may bind to kinesin-1 in a co-operative manner to form a stable ternary complex. This notion is further supported by the observation that the neurite tip localization of JIP1 is decreased by JIP3 knockdown (see Figure 3) [11]. It has been reported that JIP3 binds not only to kinesin light chain (KLC) but also to kinesin heavy chain (KHC) [22]. While we cannot rule out a possibility that JIP3–KHC binding contributes to the ternary complex formation, our results with a mutant JIP3 bearing amino acid substitution in the KLC contact site indicate that JIP3–KLC binding is essential for the ternary complex formation. As for JIP1–JIP3 binding, it has been reported that the central 189 amino acids of JIP3, which include the JBD and kinesin-1 binding regions, and the C-terminal 158 amino acids of JIP1, which include the PTB domain and kinesin-1 binding region, are sufficient for their binding [11]. Our results using JIP1-PTB domain point mutants further delineate the JIP3-binding domain in JIP1. By using domain specific mutants of JIP1 and JIP3, we showed that JIP1–JIP3–kinesin-1 ternary complex formation depends on JIP1–JIP3-binding, JIP1–kinesin-1-binding, and JIP3–kinesin-1-binding. Although the abrogation of one of these interactions prevented ternary complex formation, JIP1–JIP3 binding was independent of their binding to kinesin-1. This indicates that JIP1–JIP3 complex formation leads to the formation of the ternary complex. It has been reported that JIP1–kinesin-1 binding can trigger the activation of kinesin-1 motor activity in the presence of additional factors such as FEZ1 [23]. Therefore, JIP1–JIP3 complex formation might also be critical for the JIP1-dependent activation of kinesin-1.

Because the kinesin-1 binding activity of JIP3 is crucial for the formation of the ternary complex, other proteins that compete with JIP3 for binding to the JIP1-PTB domain and lack the capacity to bind kinesin-1 may negatively regulate the potency of JIP1 for kinesin-1 binding. Using DLK, we demonstrated a potential regulatory role for JIP1-PTB domain binding proteins in the formation of the JIP1–JIP3–kinesin-1 ternary complex. In addition, kinesin-1 or JIP3-binding proteins may also affect the formation of the JIP1–JIP3–kinesin-1 complex. The inhibition of JIP1–kinesin-1 binding by Ca2+−dependent binding of S100A6 to KLC, and the inhibition of JIP3–kinesin-1-binding by GTP-dependent binding of ARF6 to JIP3, have been reported [24,25]. Taken together, these observations suggest that kinesin-1-dependent transport is regulated by multiple signaling pathways via JIP1, JIP3 and KLC.

In this study, we have shown that JIP3 is a major binding protein of JIP1 in Neuro2a cells, and that JIP3 has the highest JIP1-binding capacity among various known JIP1-PTB domain binding proteins. Because JIP1 and JIP3 are highly expressed in neurons [26,27], these observations imply that JIP1 and JIP3 play a common role in kinesin-1 dependent intracellular transport. In fact, genetic studies in *Drosophila* and *Caenorhabditis elegans* have indicated that both JIP1 and JIP3 support vesicle transport in neural cells [4,28,29]. However, knockdown of JIP1 or JIP3 result in different phenotypes in mammalian neurons: JIP1 knockdown results in the partial inhibition of axon elongation, while JIP3 knockdown results in the stimulation of neurite elongation and branching [30,31]. In our experiments, although JIP3 was a major JIP1 binding protein in Neuro2a cells, only a fraction of the endogenous JIP3 was co-precipitated with endogenous JIP1 (see Figure 2E). This implies that a considerable fraction of JIP3 exists in protein complexes free of JIP1, and may have an additional function unrelated to JIP1. Therefore, the JIP1–JIP3 complex might not be a universal kinesin-1 regulatory module, but rather a specific regulator of intracellular transport in neuronal cells.

## Conclusions

We investigated the contribution of JIP1 binding proteins to the interaction between JIP1 and kinesin-1. The binding of JIP3 to the PTB domain of JIP1 enhanced the JIP1–kinesin-1 interaction significantly, while the binding of JNK to the JBD of JIP1 showed no significant effect. DLK, another JIP1-PTB domain binding protein, did not show any effect on JIP1–kinesin-1 binding by itself. However, the over-expression of kinase-inactive DLK perturbed the formation of the JIP1–JIP3–kinesin-1 ternary complex, suggesting that DLK might compete with JIP3 for the JIP1-PTB domain. Our results suggest that the proteins that bind to the JIP1-PTB domain function as regulators of JIP1–kinesin-1 complex formation.

## Methods

### Cells

HEK293T human embryonic kidney cells, Neuro2a mouse neuroblastoma cells, and RFP-JIP1 stably transfected Neuro2a cells (RFP-JIP1-Neuro2a) were maintained in Dulbecco’s modified Eagle’s medium supplemented with 10% fetal bovine serum (FBS). The cell lines were cultured under 5% CO_2_ at 37°C. For differentiation, Neuro2a and RFP-JIP1-Neuro2a cells were incubated with medium containing 5% FBS and 20 μM all-trans-retinoic acid (Wako) for 24–48 h.

### Transfections

For transfection, 3 × 10^5^ HEK293T or 6 × 10^5^ Neuro2a cells were plated in 6-cm dishes. After 48 h, the cells were transfected with 5–10 μg of plasmid using Lipofectamine LTX (Invitrogen). After 24 h, the cells were used for co-precipitation assays. For knock-down of JIP3, 1 × 10^6^ RFP-JIP1-Neuro2a cells were transfected with 3–5 μg of plasmids using either Nucleofector II (Amaxa) or Neon (Invitrogen) techniques, and plated in 6-cm dishes. After 48 h, the cells were used for co-precipitation assays. For immunofluorescence, Neuro2a and RFP-JIP1-Neuro2a cells were plated in 24-well plates for 24 h. The culture medium was changed to differentiation medium and the cells were incubated for a further 24 h. The cells were transfected with 0.5 μg of plasmids using Lipofectamine LTX. After 24–30 h, the cells were processed for immunofluorescence staining.

### Immunofluorescence and neurite tip measurements

Neuro2a and RFP-JIP1-Neuro2a cells were fixed with 3% paraformaldehyde in PBS for 15 min and washed three times with PBS. Following permeabilization with 0.1% Triton X-100 in PBS for 10 min, the cells were washed three times with PBS and quenched with 0.1 M glycine in PBS for 30 min. The cells were then washed a further three times with PBS and blocked with 10% calf serum in TBST for 1 h. All of these steps were performed at room temperature. The cells were then incubated in 0.1% BSA in TBST containing primary antibodies for 16–48 h at 4°C, washed three times with TBST, and labeled with Alexa 488-, 546-, or 647-conjugated anti-mouse or anti-rabbit antibodies. Quantification of neurite tip fluorescence intensity was performed using Multi Gauge software (Fujifilm). The neurite tip and its shaft were hand-selected with the rectangle tool, and the average pixel fluorescence intensity was measured. The average intensity ratio between the tip and shaft of individual neurites was employed as the localization value.

### Co-precipitation assays

All of the following steps were performed at 4°C. The cells were washed twice with ice-cold PBS and lysed in 0.5 ml of lysis buffer (150 mM NaCl, 20 mM Hepes, pH 7.5, 1 mM EGTA, 50 mM NaF, 25 mM beta-glycerophosphate, 1 mM Na_3_VO_4_, pH 10, 5 μg/ml aprotinin, 10 μg/ml leupeptin, 0.5% Triton X-100, 1% protease inhibitor cocktail (Sigma)) for 10 min on ice. The lysates were then centrifuged at 15,000 × g for 20 min, and the resulting supernatants were used for co-precipitations. The supernatants were incubated with either 1 μg of antibody or Streptavidin Sepharose (GE Healthcare) for 90 min, following which the immunocomplexes were recovered with Protein A Sepharose (BD Biosciences Pharmingen). The Streptavidin Sepharose and Protein A Sepharose pellets were then washed three times with lysis buffer and boiled with SDS sample buffer.

Two whole brains from ICR E17 mouse embryos were Dounce-homogenized 10 times in 1 ml of lysis buffer and incubated on ice for 10 min. The homogenate was then centrifuged at 15,000 × g for 20 min, and the resulting supernatants were used for co-precipitations. The supernatants were incubated with either 8 μg of anti-JIP1 antibody or normal rabbit IgG for 4 hours, following which the immunocomplexes were recovered with Protein A Sepharose. The Protein A Sepharose pellets were then washed three times with lysis buffer and boiled with SDS sample buffer.

### JIP1, JIP3, KLC, KHC, APP, MEKK1 and DLK expression vectors

The mouse JIP-1b cDNA (encoding a polypeptide of 707 aa) was cloned into the pEGFP-C vector (Clontech) and the pNTAP vector (Stratagene) (GFP-JIP1-WT and TAP-JIP1-WT, respectively). GFP-JIP1-WT was digested with BglII and self-ligated to generate the GFP-JIP1-dJBD vector. The GFP-JIP1-WT and TAP-JIP1-WT vectors were digested with EcoRV and ligated with a stop codon linker to generate the GFP-JIP1-dCT and TAP-JIP1-dCT vectors. As the first step in the generation of the GFP-JIP1-V581A vector, the JIP1-WT vector was digested with StuI and EcoRV, and the fragment was sub-cloned into pBlueScript KS (JIP1-PTB-pBS). Silent mutations were introduced by site-directed mutagenesis PCR to generate BsrGI and NruI sites at 571 and 590, respectively. The synthetic complementary oligonucleotides containing the V581A mutation were cloned into the BsrGI-NruI site. Then, the resultant vector was digested with BstEII and EcoRV, and the fragment was cloned into GFP-JIP1-WT. GFP-JIP1-F642V and F687V were produced by site-directed mutagenesis PCR. GFP-JIP1-V581A, -F642V and -F687V mutants were digested with BstEII and EcoRV, and the fragments were cloned into the TAP-JIP1-WT vector. Mouse JIP3-c (1337aa) was cloned into pEGFP and pNTAP vectors (GFP-JIP3-WT and TAP-JIP3-WT, respectively). GFP-JIP3-LZ4A was produced by site-directed mutagenesis PCR. GFP-JIP3-LZ4A was digested with HindIII and BglII, and the fragment was cloned into the TAP-JIP3-WT vector (TAP-JIP3-LZ4A). Mouse KLC-1d (608 aa) and KHC (KIF5C) (957 aa) were cloned into the pCMV-V5 vector (V5-KLC and V5-KHC, respectively). The T7-MEKK1, T7-DLK-WT and T7-DLK-KR vectors were described previously [15,32]. The cDNA encoding human APP (APP695) was cloned into the pEGFP-N vector. The cytoplasmic region of APP (comprising amino acids 649–695) was produced by PCR and cloned into the pEGFP-C vector (GFP-APPC).

### Short hairpin RNA expression vectors

A shRNA (short hairpin RNA) plasmid targeting mouse JIP3 was constructed using DNA oligos designed with a 19-mer sense sequence, a nine-nucleotide loop, and a 19-mer antisense sequence (sense: 5′-gatccccGGTCTTG ACCCAACATGAAttcaagagaTTCATGTTGGGTCAAGA CCttttta-3′, and antisense: 5′-tcgataaaaaGGTCTTGACC CAACATGAAtctcttgaaTTCATGTTGGGTCAAGACCG GG-3′). Annealed oligos were cloned into the BglII and SalI sites of the pSuper.gfp/neo vector (OligoEngine).

### Antibodies

The following antibodies were used: anti-JIP1 mAb (TDL), anti-JIP1 pAb (Zymed), anti-KHC mAb (Chemicon), anti-JIP3 pAb (Santa Cruz), anti-DLK pAb (described previously [33]), anti-JNK1 mAb (BD Biosciences Pharmingen), anti-JNK2 mAb (Santa Cruz), anti-GFP mAb (Santa Cruz), anti-GFP pAb (MBL), anti-RFP pAb (Clontech), anti-V5 mAb (Invitrogen), anti-SBP mAb for TAP-tagged proteins (Santa Cruz), anti-Omni mAb for T7-DLK (Santa Cruz), HRP-conjugated anti-rabbit IgG and anti-mouse IgG (GE Healthcare), HRP-conjugated anti-goat IgG (Zymed).

### Western blotting

Proteins were separated by SDS-PAGE and analyzed by western blotting according to standard protocols. Horseradish peroxidase-conjugated secondary antibodies were used to detect bound primary antibodies. Signal intensities were quantified from the original data recorded by a LAS-4000 Multi Luminescent Image Analyzer and Multi Gauge software (Fujifilm).

### Mass spectrometry analysis

Neuro2a cells (~5 × 10^7^ cells) were transfected with the TAP-JIP1 expression vector. One day after transfection, retinoic acid (all-trans, Sigma) was added to the culture medium to a final concentration of 20 μM. Cells were further cultured for 2 days before lysing in 6 ml of the lysis buffer used for the co-precipitation assays (see above). The cell lysate was clarified by centrifugation at 100,000 × g for 30 min, and TAP-JIP1 binding protein was collected as described for the co-precipitation assays. The TAP-JIP1 binding proteins were separated by SDS-PAGE and detected by silver staining for imaging or by SYPRO Ruby (Molecular Probes) for mass spectrometry. The band of interest was cut out and digested with trypsin. The eluted peptides were analyzed on a 4800 MALDI TOF/TOF system (Applied Biosystems).

## Abbreviations

JIP: c-Jun N-terminal kinase (JNK) interacting protein;PTB: Phosphotyrosine binding;KHC: Kinesin heavy chain;KLC: Kinesin light chain;APP: Amyloid precursor protein;DLK: Dual leucine zipper bearing kinase;WT: Wild type

## Competing interests

The authors declare that they have no competing interests.

## Authors’ contributions

TS and SH designed the experiments. TS and KO performed and analyzed the immunoprecipitation assays. TS performed and analyzed the immunofluorescence assays. HH and YY performed the mass spectrometry analysis. TS, KO, YB, JS and SH constructed the expression vectors. TS, SH and SO wrote the manuscript. All authors read and approved the final manuscript.

## Supplementary Material

Additional file 1: Figure S1 JNK activity does not affect JIP1 binding to kinesin-1. (**A**). Co-precipitation of GFP-JIP1 mutants and endogenous JNK in Neuro2a cells. The same samples as shown in Fig. 2A were analyzed by WB with the indicated antibodies. (**B**). Neuro2a cells transiently expressing TAP-JIP1 were treated with SP600125 (20 μM) or DMSO (vehicle) for 2 hours. Lysates were precipitated with SA. *SA*, proteins bound to streptavidin sepharose. (**C**). RFP-JIP1-Neuro2a cells were transfected with expression vectors for T7-MEKK1 or pUC8 (empty vector). Lysates were immunoprecipitated with anti-RFP antibody and analyzed by WB with the indicated antibodies. *: non-specific band. Click here for file

Additional file 2: Figure S2 Sequence alignments of the JIP1 PTB domain. (**A**) JIP1 PTB domain aligned with Shc- and Dab1-PTB domains. All sequences are from mouse. The gray shading indicates the amino acids corresponding to JIP1 V581, F642 and F687. (**B**). Alignment of JIP1 PTB domains between species. The gray shading indicates the amino acids corresponding mouse JIP1 V581, F642 and F687. Click here for file

Additional file 3: Figure S3 Mouse JIP3 peptides identified by Mascot search (Matrix Science). Amino acid sequence of mouse JIP3. Capital letters shaded gray indicate the peptides identified by Mascot search. Click here for file

Additional file 4: Figure S4 The effect of JIP3 knockdown was offset by JIP3-WT but not JIP3-LZ4A. (**A**). Lysates prepared from RFP-JIP1-Neuro2a cells expressing shRNA targeting JIP3 (shJIP3), non-silencing control (NS) shRNA, TAP-JIP3-WT, or TAP-JIP3-LZ4A, in the combination as indicated were immunoprecipitated with anti-RFP antibody and analyzed by WB with the indicated antibodies. Input, cell lysate used for the immunoprecipitation assay. IP:RFP, immunoprecipitated proteins. (**B**). Quantification of kinesin-1 binding by RFP-JIP1 in (A). Kinesin-1 binding was normalized to the amount of precipitated RFP-JIP1 and input of kinesin-1. Results of two independent experiments are shown. (**C**). Differentiated RFP-JIP1-Neuro2a cells were transfected with shRNA vectors (NS or shJIP3) containing a GFP expression cassette. Arrowheads indicate the neurite tips of transfected cells. Scale bar = 20 μm. (**D**). Quantification of the relative fluorescence of RFP-JIP1 in the neurite tip. *: p < 0.03. Error bars indicate ± SEM. n = 50 for each construct. Click here for file
