# Supplementary figures and images for: The interaction of Kinesin-1 with its adaptor protein JIP1 can be regulated via proteins binding to the JIP1-PTB domain
Source: BMC Cell Biol. 2013 Mar 4;14:12. doi: 10.1186/1471-2121-14-12 (PMC3599065; doi:10.1186/1471-2121-14-12)

Supplemental figure 1

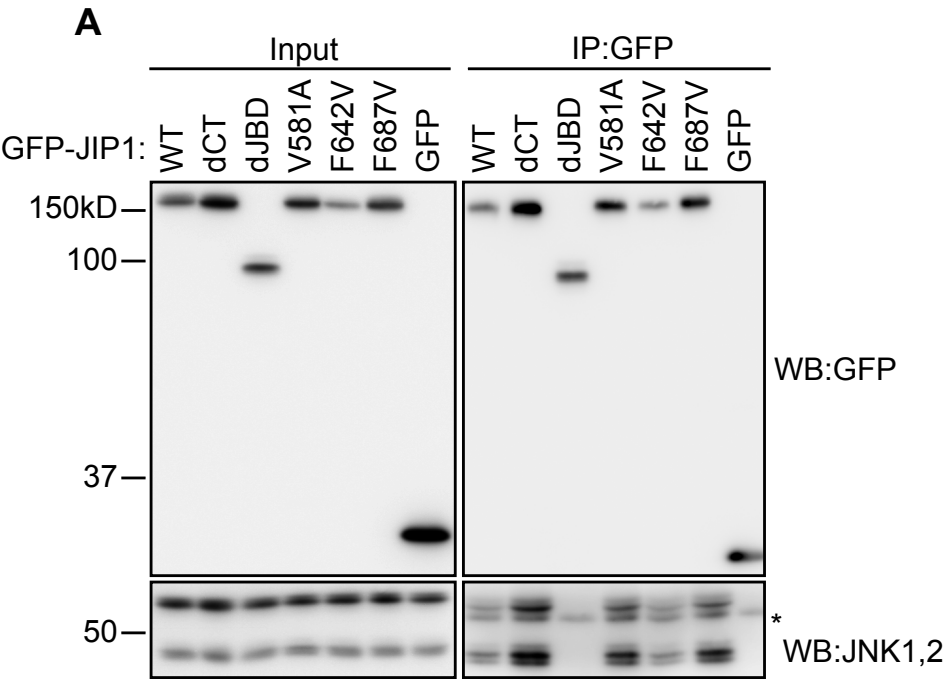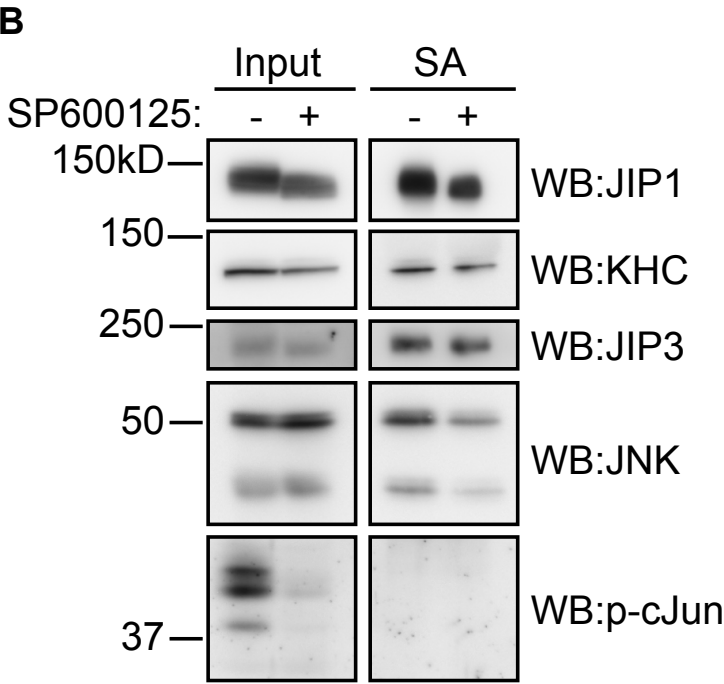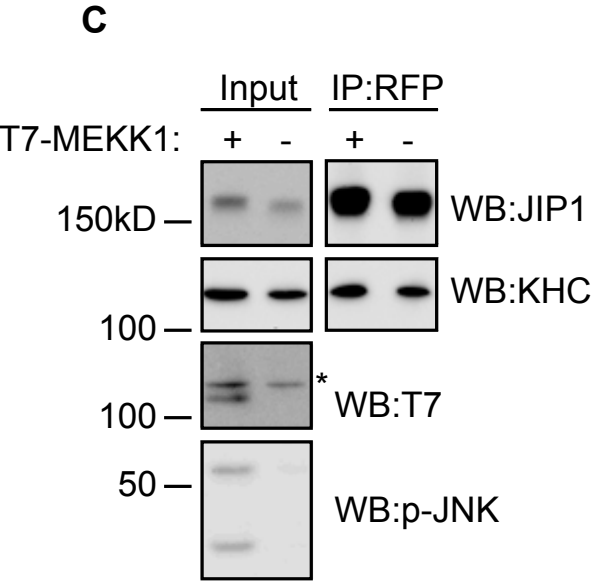

Supplement: Additional file 1: Figure S1 — JNK activity does not affect JIP1 binding to kinesin-1. (A). Co-precipitation of GFP-JIP1 mutants and endogenous JNK in Neuro2a cells. The same samples as shown in Fig. 2A were analyzed by WB with the indicated antibodies. (B). Neuro2a cells transiently expressing TAP-JIP1 were treated with SP600125 (20 μM) or DMSO (vehicle) for 2 hours. Lysates were precipitated with SA. SA, proteins bound to streptavidin sepharose. (C). RFP-JIP1-Neuro2a cells were transfected with expression vectors for T7-MEKK1 or pUC8 (empty vector). Lysates were immunoprecipitated with anti-RFP antibody and analyzed by WB with the indicated antibodies. *: non-specific band. [file 1471-2121-14-12-S1.pdf]
