# Supplementary material for: The interaction of Kinesin-1 with its adaptor protein JIP1 can be regulated via proteins binding to the JIP1-PTB domain
Source: BMC Cell Biol. 2013 Mar 4;14:12. doi: 10.1186/1471-2121-14-12 (PMC3599065; doi:10.1186/1471-2121-14-12)
Supplement: Additional file 3: Figure S3 — Mouse JIP3 peptides identified by Mascot search (Matrix Science). Amino acid sequence of mouse JIP3. Capital letters shaded gray indicate the peptides identified by Mascot search. [file 1471-2121-14-12-S3.pdf]

Supplemental figure 3

1 mmeiqmdegg gvvvyqddyc sgsvmserVS GLAGSIYREF ERIihcydee vvkelmplvv
61 nvlenldsvl senqehevel ellredneql ltqyerekal rkqaeekfie fedaleqekk
121 ELQIQVEHYE FQTRqlelka knyadqisrl eeresemkke ynalhqrhte miqtyvehie
181 rskmqqvggs gqtesslpgr srkerptsln vfpladgmvr aqmggklvpa gdhwhlsdlg
241 qlqssssyqc pndemsesgq ssaaatpstt gtksntptss vpsaavtpln eslqplgdyv
301 svtknnkqar ekrnsrnmev qvtqemrnvs igmgssdews dvqdiidstp eldvcpetrl
361 ertgssptqg ivnkafgint dslyhelsta gsevigdvde gadllgefsv rddffgmgke
421 vgnlllensq lletknalnv vkndliakvd qlsgeqevlk geleaakqak vklenrikel
481 eeelkrvkse avtarrepre evedvssylc teldkipmaq rrrftrvema rvlmernqyk
541 erlmelqeav rwtemirasr ehpsvqekkk stiwqffsrl fssssspppa krSYPSVNIH
601 YKSPTAAGFS QRrshalcqi sagsrpleff pdddctssar reqkreqyrq vrehvrnddg
661 rlqacgwslp akykqlspng gqedtrmknv pvpvycrplv ekdpstklwc aagvnlsgwk
721 pheedssngp kpvpgrdplt cdregegepk sthpspekkk aketpeadat ssrvwiltst
781 lttskvviid anqpgtivdq ftvcnahvlc issipaasds dyppgemfld sdvnpedsga
841 dgvlagitlv gcatrcnvpr sncssrgdtp vldkgqgdva ttangkvnps qsteeateat
901 evpdpgpses eattvrpgpl tehvftdpap tpssstqpas engsesngti vqpqvepsge
961 lstttssaap tmwlgaqngw lyvhsavanw kkclhsiklk dsvlslvhvk grvlvaladg
1021 tlaifhrged gqwdlsnyhl mdrghphhsi rcmavvndrv wcgyknkvhv iqpktmqiek
1081 sfdahprres qvrqlawigd gvwvsirlds tlrlyhahth qhlqdvdiep yvskmlgtgk
1141 lgfsfvrita lliagnrlwv gtgngvvisi pltetvvlhr gqllglrank tsptsgegtr
1201 pggiihvygd dssdkaassf ipycsmaqaq lcfhghrdav kffvsvpgnv latlngsvld
1261 spsegpgpaa paadaegqkl kNALVLSGGE GYIDFRigdg eddeteecag dvnqtkpsls
1321 kaershiivw qvsytpe
