# Supplementary material for: The interaction of Kinesin-1 with its adaptor protein JIP1 can be regulated via proteins binding to the JIP1-PTB domain
Source: BMC Cell Biol. 2013 Mar 4;14:12. doi: 10.1186/1471-2121-14-12 (PMC3599065; doi:10.1186/1471-2121-14-12)

Supplemental figure 4

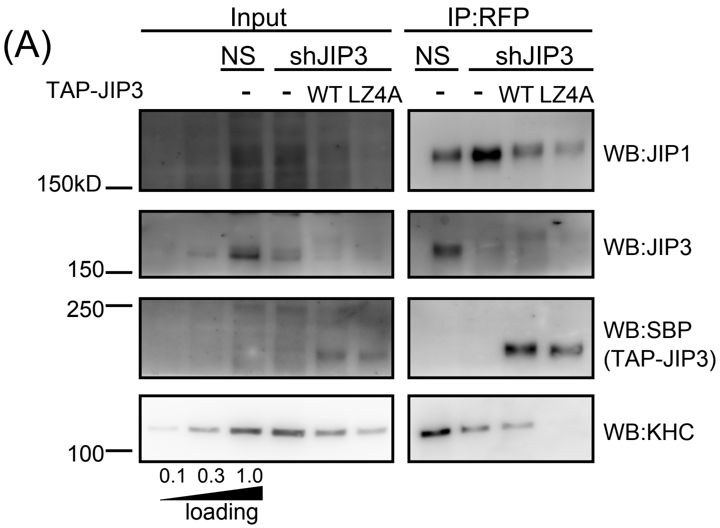

(B)

Relative intensity of bound kinesin-1

	NS	shJIP3	shJIP3+TAP-JIP3: JIP3-WT	shJIP3+TAP-JIP3: JIP3-LZ4A
Experiment 1	1.00	0.47	1.02	0.40
Experiment 2	1.00	0.24	0.62	0.30

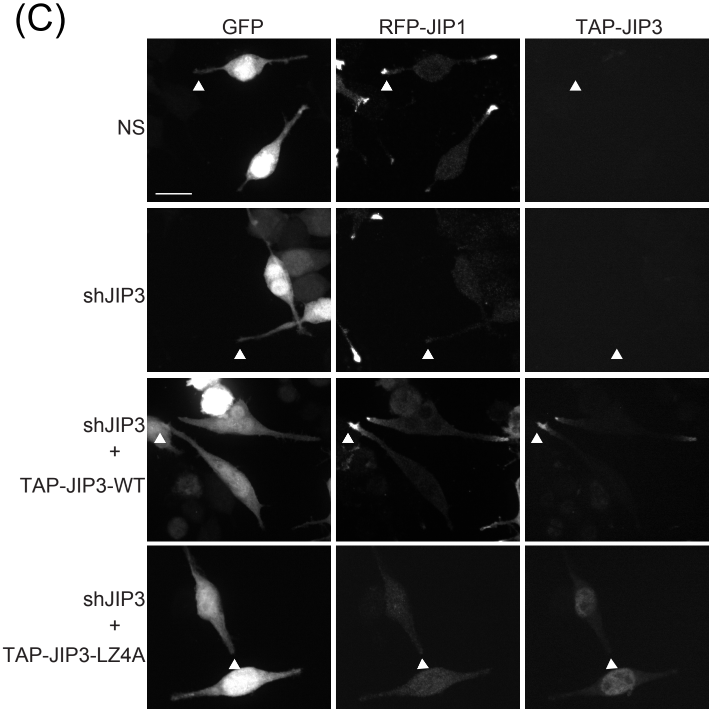

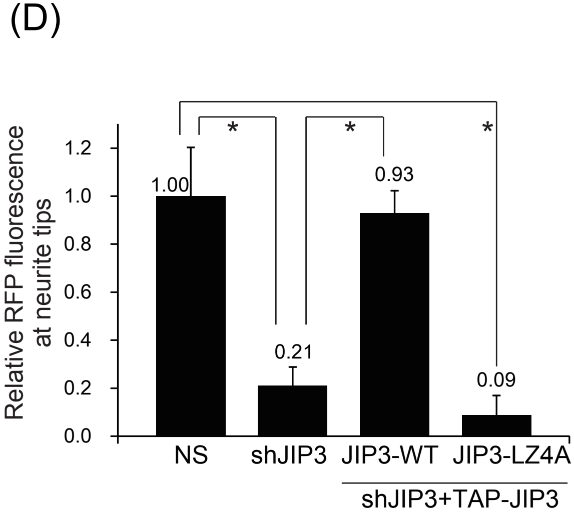

Supplement: Additional file 4: Figure S4 — The effect of JIP3 knockdown was offset by JIP3-WT but not JIP3-LZ4A. (A). Lysates prepared from RFP-JIP1-Neuro2a cells expressing shRNA targeting JIP3 (shJIP3), non-silencing control (NS) shRNA, TAP-JIP3-WT, or TAP-JIP3-LZ4A, in the combination as indicated were immunoprecipitated with anti-RFP antibody and analyzed by WB with the indicated antibodies. Input, cell lysate used for the immunoprecipitation assay. IP:RFP, immunoprecipitated proteins. (B). Quantification of kinesin-1 binding by RFP-JIP1 in (A). Kinesin-1 binding was normalized to the amount of precipitated RFP-JIP1 and input of kinesin-1. Results of two independent experiments are shown. (C). Differentiated RFP-JIP1-Neuro2a cells were transfected with shRNA vectors (NS or shJIP3) containing a GFP expression cassette. Arrowheads indicate the neurite tips of transfected cells. Scale bar = 20 μm. (D). Quantification of the relative fluorescence of RFP-JIP1 in the neurite tip. *: p < 0.03. Error bars indicate ± SEM. n = 50 for each construct. [file 1471-2121-14-12-S4.pdf]
