# Supplementary material for: The interaction of Kinesin-1 with its adaptor protein JIP1 can be regulated via proteins binding to the JIP1-PTB domain
Source: BMC Cell Biol. 2013 Mar 4;14:12. doi: 10.1186/1471-2121-14-12 (PMC3599065; doi:10.1186/1471-2121-14-12)
Supplement: Additional file 2: Figure S2 — Sequence alignments of the JIP1 PTB domain. (A) JIP1 PTB domain aligned with Shc- and Dab1-PTB domains. All sequences are from mouse. The gray shading indicates the amino acids corresponding to JIP1 V581, F642 and F687. (B). Alignment of JIP1 PTB domains between species. The gray shading indicates the amino acids corresponding mouse JIP1 V581, F642 and F687. [file 1471-2121-14-12-S2.pdf]

Supplemental figure 2

A

```
                                 JIP1 V581A
                                     ↓
JIP1  ----------QFRVKFLGSVQV-------PYHKGNDVLCAAMQKIAT-TRRLTVHFN--------------------PPSSCVL
Shc   KV---MGPGVSYLVRYMGCVEVLQSMRALDFNTRTQVTREAISLVCEAVPGAKGATRRRKPCSRPLSSILGRSNLKFAGMPITL
Dab1  LIKRFKGEGVRYKAKLIGIDEV-------SAARGDKLCQDSMMKLKGVVAGARSKGE--------------------HKQKIFL

JIP1  EISVRGVKIGVKADDALEAKGNKCSHFFQLKNISFC-GYHPKNNKYFGFITKHPADHRFACHVFVSEDSTKALAESVGRAFQQFYKQFVE
Shc   TVSTSSLNLMAA-------DCKQIIANHHMQSISFASGGDPDTAEYVAYVAKDPVNQRACHILECPEGLAQDVISTIGGQAFELRFKQY--
Dab1  TISFGGIKIFDE-------KTGALQHHHAVHEISYI-AKDITDHRAFGYVCGKEGNHRFVA--IKTAQAAEPVILDLRDLFQLIYELKQR
                                   ↑                                               ↑
                               JIP1 F642                                       JIP1 F687
```

B

```
                                    V581A
                                      ↓
C.elegans         TFYLTMLASIEVAHHKGNDVLTQAMNKVLSMYKNSEEIIVPQTVLMEISFRGIHVIDKRRKNFFQ---CPMFDF
D.melanogaster    RYLLGYLGSVETLAHKGTGVVCQAVRKIVGEYGNSP---TGQTCILEVSDQGLRMVDRSGPNQNKKDKKPCIDY
M.musculus        QFRVKFLGSVQVPYHKGNDVLCAAMQKIATTRRLTVHFNPPSSCVLEISVRGVKIGVKADDALEAKG-NKCSHF
H.sapiens         QFRVKFLGSVQVPYHKGNDVLCAAMQKIATTRRLTVHFNPPSSCVLEISVRGVKIGVKADDSQEAKG-NKCSHF

C.elegans         FYSLQNISFCGAHPKQLKYFGFITKHPLLPRFACHVFMSKNTTQPIVEAIGRAFKRSYDEYMAFAHPTEDIYLE
D.melanogaster    FYSLKNVSFCAFHPRDHRFIGFITKHPTVQRFACHVFKGSESTRPVAEAVGRAFQRFYQKFIETAYPIEDIYIE
M.musculus        F-QLKNISFCGYHPKNNKYFGFITKHPADHRFACHVFVSEDSTKALAESVGRAFQQFYKQFVEYTCPTEDIYLE
H.sapiens         F-QLKNISFCGYHPKNNKYFGFITKHPADHRFACHVFVSEDSTKALAESVGRAFQQFYKQFVEYTCPTEDIYLE
                          ↑                                            ↑         ---------
                        F642V                                        F687V     Kinesin-1 binding
                                                                               required
```
